# Blood Pressure Variability and Complexity in Nursing Home Residents Before Death

**DOI:** 10.1093/geroni/igab046.532

**Published:** 2021-12-17

**Authors:** Laura Graham, Sei Lee, Michael Steinman, Carmen Peralta, Anna Rubinsky, Bocheng Jing, Kathy Fung, Michelle Odden

**Affiliations:** 1 VA Palo Alto Health Care System, Menlo Park, California, United States; 2 University of California San Francisco, San Francisco, California, United States; 3 University of California, San Francisco, San Francisco, California, United States; 4 San Francisco VA Medical Center, San Francisco, California, United States; 5 Stanford University, Stanford, California, United States

## Abstract

Blood pressure (BP) is a complex dynamic system in the human body and an important determinant of healthy aging. Exploring BP as a dynamic data system may provide important insights into how BP patterns can provide complementary information to the static, one-time BP measurements that are more commonly used for clinical decision making. Thus, we sought to describe BP as a dynamic data system in older adults nearing death. Using a prospective cohort study design, we assessed BP measures 6 months before death in Veterans Health Administrative nursing home residents between 10/1/2006 and 9/30/2017. Variability was characterized using standard deviation and mean square error after adjusting for diurnal variations. Complexity (i.e., amount of novel information vs. redundancy) was examined using Shannon’s entropy (bits). Generalized linear models were used to examine factors associated with overall BP variability. We identified 17,953 patients (98.0% male, 82.5% White, mean age 80.2 years, and mean BP 125.7/68.6 mmHg). In the last 6 months of life, systolic BP decreased slightly (□-7.2mmHg). Variability was stable until the last month of life, at which point variability increased by as much as 30%. In contrast, complexity did not change in the 6 months before death (□0.02 bits). Factors associated with BP variability before death include hospitalizations, hospice care, and medication changes. Systolic BP decreases in the last 6 months before death, and BP variability increases in the last month of life. Further, the increase in BP variability may be driven by increasingly complex care patterns as one approaches death.

